# Preparation and functional validation of rabbit anti-canine CD3ε monoclonal antibody

**DOI:** 10.3389/fvets.2025.1612069

**Published:** 2025-12-04

**Authors:** Huixin Li, Mengjuan Chen, Rongmiao Yue, Sihao Li, Bowen Shi, Mengke Qin, Qingda Meng, Shanshan Xie

**Affiliations:** 1 College of Veterinary Medicine, Henan Agricultural University, Zhengzhou, China; 2 Research Center for Organoids, College of Veterinary Medicine, Henan Agricultural University, Zhengzhou, China; 3 Henan Ruipai Xiaoqiaopi Pet Hospital, Zhengzhou, China; 4 Department of Cell Biology and Genetics, Chongqing Medical University, Chongqing, China

**Keywords:** canine, CD3, monoclonal antibody, immunotherapy, animal model

## Abstract

**Introduction:**

CD3, a surface antigen critical for T cell activation and signal transduction, serves as a key diagnostic marker for T cell-associated malignancies and a therapeutic target in immunotherapy. With canine models gaining prominence in translational immunology and oncology, reliable tools to study T cell-mediated immunity are essential.

**Methods and Results:**

In this study, recombinant canine CD3ε protein was generated via eukaryotic expression and used to immunize rabbits, yielding a novel rabbit anti-canine CD3ε monoclonal antibody (mAb), designated HORCF-CD3.1. Functional characterization confirmed its specificity through flow cytometry and Western blot, demonstrating robust binding to CD3 molecules. Furthermore, the mAb effectively induced T cell stimulation *in vitro* when applied as an anti-CD3 activator. These findings validate HORCF-CD3.1 as a versatile tool for ELISA, Western blot, and flow cytometry applications.

**Discussion:**

The successful development of this species-specific antibody provides a foundation for advancing diagnostic and therapeutic strategies targeting T cell-related diseases in both humans and dogs.

## 1 Introduction

In immunological research and clinical diagnosis, the function and regulatory mechanism of T cells, as an important component of the adaptive immune system, have been a focus of research. Binding of the T cell receptor (TCR) to the CD3 complex is essential for T cell recognition and activation. The CD3 molecule, an important differentiation antigen located on the T cell membrane, is a protein complex that is expressed on the surface of all mature T cells (1). It consists of five peptide chains, *γ*, *δ*, *ε*, *ζ*, and *η*, all of which are transmembrane proteins. Among these, CD3ε serves as a critical component. During T cell activation, the immunoreceptor tyrosine activation motif (ITAM) in the cytoplasmic structural domain of CD3ε is phosphorylated by Scr family kinases, thereby triggering the activation of downstream signaling pathways (2). The primary function of CD3 is to transduce activation signals generated by TCR recognition of antigens into T cells, thus playing a central role in the T cell-mediated immune response. Since CD3ε plays a key role in T cell activation, the use of bispecific antibodies against CD3ε and tumor-associated antigen (TAA) has been a focus of research in tumor therapy. For example, blinatumomab is a bispecific antibody targeting CD3ε and CD19, which is used for the treatment of acute B-lymphoblastic leukemia (BLL) (3).

As important companion animals for humans, pet dogs are characterized by a higher incidence of spontaneous tumors, having a five-fold greater chance of developing spontaneous cancers than humans (4). Canine spontaneous tumors cover a wide range of types, including B-cell lymphomas, osteosarcomas, and melanomas, among others (5). Traditional treatments for canine tumors include surgical therapy, radiation therapy, and chemotherapy, which, while improving the survival and prognosis of diseased dogs to some extent, still suffer from high side effects, as well as the potential for immune escape and mutation of tumor cells (6, 7). Thus, novel treatments are needed, whereas tumor immunotherapy offers hope for canine tumor treatment. The development of CD3ε monoclonal antibodies serves as a critical tool for studying canine T-cell biology and pathology. Their recognition of individual epitopes of target antigens has high specificity and strong affinity, making them valuable in diagnostic and therapeutic applications. However, current research on canine CD3ε is relatively limited, especially regarding its function and regulatory mechanisms in disease states. Therefore, the development and validation of a monoclonal antibody to canine CD3ε is important for understanding the immunoregulation of canine T cells and its role in immunoregulation.

Our study aims to develop and validate a canine CD3ε monoclonal antibody to provide a new tool for canine T cell-related research. This antibody can not only be applied to the *in vitro* expansion and functional studies of T cells, but also serve as a critical component of bispecific antibodies in immunotherapy and tumor-targeted therapy, among other fields. In future studies, this antibody will serve a dual purpose: as a diagnostic tool for lymphoma, and as a foundational component for constructing bispecific antibodies for the treatment of canine cancers. Furthermore, it will be utilized as a critical reagent to screen for novel immune checkpoint inhibitors, thereby paving the way for advanced combination immunotherapies.

## 2 Materials and method

### 2.1 Ethics statement

Approved by the Ethics Committee for Laboratory Animal Care (Animal Ethics Procedures and Guidelines of China) at the Henan Agricultural University (Permit No. HNND2023031013).

### 2.2 Canine CD3ε plasmid construction

Based on the full amino acid sequence of canine CD3ε protein (P27597) provided by Uniprot,^1^ the 181-amino-acid segment from positions 22–202 at the N-terminus was selected as the antigenic peptide. For eukaryotic protein expression, a plasmid was synthesized by linking this antigenic peptide to the human—Fc tag. Meanwhile, for prokaryotic protein expression, another plasmid was constructed by fusing the same 181-amino-acid antigenic peptide with the His tag.

### 2.3 Transformation, induced expression and purification of canine CD3ε recombinant plasmid

For eukaryotic expression, the CD3ε -Fc gene construct was cloned into a mammalian expression vector. The recombinant plasmid was first propagated in *Escherichia coli* JM108 competent cells, and transformants were selected on ampicillin-containing agar plates. The plasmid was then extracted from a single colony using a standard miniprep kit (Cat#DP106, Tiangen, China). For mammalian cell transfection, the purified plasmid DNA was complexed with a transfection reagent Lipofectamine 2000 (Cat# 11668019, Thermofisher, America) and introduced into CHO (Chinese Hamster Ovary) cells. The culture was maintained for 5 days to allow for protein expression and secretion. After the cells were harvested and lysed using a non-denaturing lysis buffer (50 mM Tris, 300 mM NaCl [Cat# LYHG22201, chemreagent, China], 20 mM Imidazole [Cat# I8090, Solarbio, China], pH 8.0) to preserve the native structure of the protein. Subsequent protein purification was performed with Protein A Agarose Beads/Resin (Cat# 10600-P07E-RN, Sino Biological, China) following the manufacturer’s instructions.

The gene fragment encoding the target protein (canine CD3ε) with a C-terminal His-tag was synthesized and cloned into a pET expression vector. The recombinant plasmid was transformed into *E. coli* BL21 competent cells for protein expression. A single positive colony was selected to inoculate a liquid culture. When the culture reached the mid-logarithmic growth phase, protein expression was induced by the addition of 0.5 mM Isopropyl *β*-D-1-thiogalactopyranoside (IPTG; Cat# I8070, Solarbio, China) and continued for 20 h at 16 °C. The bacterial were then harvested by centrifugation. The bacterial pellet was resuspended in PBS (Cat#P1020, Solarbio, China) and subjected to ultrasonic disruption on ice. Following centrifugation, the target protein was found primarily in the form of inclusion bodies within the pellet. The inclusion bodies were solubilized in a denaturing buffer containing 6 M Urea (Cat# A510907-0500, Sangon Biotech, China).

### 2.4 Animal immunization

After emulsifying the purified CD3ε-Fc protein with Freund’s adjuvant (complete adjuvant for the first immunization and incomplete adjuvant for subsequent immunizations) (Cat#P2301, P2306, Beiotime, China) in equal volumes, New Zealand white rabbits were injected subcutaneously at multiple points on the back. For the first immunization, 0.5 mg of the antigen was used. Fourteen days later, for the booster immunization, 0.25 mg of the antigen was emulsified with an equal volume of Freund’s incomplete adjuvant and then injected. Subsequently, immunizations were carried out once every 7 days, for a total of 4 times. Seven days after the last immunization, blood was collected from the marginal ear vein. After centrifugation, the serum was collected and antibody titers were measured using indirect ELISA. Subsequent experiments were initiated upon confirmation of antibody titers reaching 1:100,000.

### 2.5 Isolation of the memory B cells (MBCs)

Preliminary screening was conducted with the B-cell surface markers (CD19+/IgD-/CD27+). The immunizing protein and the anti-IgG Fc secondary antibody were labeled with distinct fluorophores. Memory B cells (MBCs) expressing surface IgG antibodies that specifically bound to the antigen were thus dual-fluorescent. Finally, these target MBCs were sorted from the cell suspension using a flow cytometer (BD FACS, America) and deposited into a 96-well culture plate.

### 2.6 Amplification, sequencing of heavy and light chains of CD3ε monoclonal antibody and antibody expression

B cells were lysed at room temperature, and the single-cell antibody gene amplification system was used to obtain the full sequence of the light chain and the Fab fragment of the heavy chain, respectively. The amplified target heavy-chain gene was connected to the pcDNA3.4 vector containing the rabbit IgG Fc fragment, and the light-chain gene to the blank pcDNA3.4 vector to construct a dual-plasmid system. Subsequently, the HEK293 cells were transiently transfected with the dual-plasmid system for antibody expression. After 5 days, the supernatant was collected for detection. Following successful validation, the antibody was produced at a larger scale. The expressed supernatant was dialyzed against PBS buffer at 4 °C and subsequently purified using rProtein A Beads (Cat# SA012005, smart-lifesciences, China). The final product, a monoclonal antibody against CD3ε (HORCF-CD3.1).

### 2.7 Identification of the reactivity of CD3ε monoclonal antibody

Canine CD3ε-Fc protein (2 μg/mL) was coated on ELISA plate (Nunc Thermofisher) before blocking with 0.1% BSA (bovine serum albumin) in PBS with 0.05% Tween 20. Supernatants from HEK293 cells transfected with the antibody gene were served as the primary antibody. HRP-conjugated goat anti-rabbit IgG (Cat# AS104, Abclonal, China) was used as the secondary antibody, human IgG Fc fragment and feline CD3ε-His recombinant protein as the antigen-negative controls, wild-type HEK293 cell supernatant as the antibody-negative control, and serum from rabbits immunized with CD3-Fc fusion protein as the positive control.

### 2.8 Immunoblotting

Protein(CD3ε-Fc) transfer from the SDS-PAGE gel to a polyvinylidene difluoride membrane (PVDF) (Cat# ISEQ00010, Sigma, America) was performed using a rapid transfer system (Trans-Blot Turbo Transfer System, Bio-Rad, Australia) at 400 mA, for 25 min. The produced monoclonal antibody was used as the primary antibody and incubated overnight at 4 °C. Afterwards, the membrane was incubated with a horseradish peroxidase-conjugated secondary antibody. Chemiluminescent and colorimetric settings (Bio-Rad, Australia) were used to image the membranes.

### 2.9 Isolation of canine PBMCs and culture of T cells

Canine peripheral blood mononuclear cells (PBMCs) were isolated with Ficoll Plus 1.077 (Cat#P4350, Solarbio, China) from freshly collected whole blood, then cultured in RPMI 1640 medium with 10% fetal bovine serum (FBS) and 10 ng/mL interleukin—2 (IL-2) (Cat#C147S, MedChemExpress, American) and incubated at 37 °C with 5% CO_2_. The cells were observed daily for sub-culture and cryopreservation when needed.

### 2.10 Evaluation of the CD3ε monoclonal antibody binding affinity by flow cytometry

The binding affinity of the produced CD3ε monoclonal antibody was assessed by flow cytometry using both cultured T cells and cryopreserved PBMCs from our laboratory. Cells were stained with this antibody (primary antibody) followed by an Alexa Fluor 488-conjugated goat anti-rabbit secondary antibody (Cat#AS053, abclonal, China). After a 20 min incubation at 4 °C, the cells were washed, resuspended in flow buffer, and filtered through a cell strainer. Samples were kept on ice until acquisition on a FACS Calibur flow cytometer (Becton Dickinson). Subsequent data analysis was performed with FlowJo V10.8.1.

### 2.11 T cell stimulation via CD3 cross-linking

Cultured canine T cells were plated in a 96-well plate at 1 × 10^5^ cells per well in a final volume of 200 μL. Stimulation was initiated by adding the canine CD3ε monoclonal antibody to a final concentration of 10 ng/mL. After 48-h incubation, the cell supernatant was carefully collected for the detection of the canine IFN-*γ* release using a standardized canine IFN-γ enzyme-linked immunosorbent assay (ELISA) kit (Cat# 3113-1H-20, Mabtech, Sweden), following the manufacturer’s instructions.

### 2.12 Generation of CMT-U27 cells stably expressing membrane-bound anti-CD3 scFv (anti-CD3-CMT-U27)

A stable CMT-U27 cell line expressing membrane-anchored anti-canine CD3 scFv was generated as previously described (8). Briefly, the heavy and light chains of monoclonal antibody, HORCF-CD3.1, were assembled as a single construct and separated by a flexible linker composed of Glycine and Serine (VL-linker-VH). For membrane targeting, an N-terminal CD8α signal peptide (Uniprot: P01732.1: 1-21aa) was added, followed by a Myc tag (EQKLISEEDL) to assess membrane surface expression. To facilitate plasma membrane anchoring, a C-terminal PDGFR transmembrane domain (Uniprot: Po9619.1512-561aa) was incorporated into the scFv construct. Membrane-bound anti-CD3 scFv was ultimately cloned into pLVX-EF1α-IRES-Puromycin. The construct was transduced into CMT-U27 cells via lentiviral vectors, and the resulting cell line was designated as anti-CD3-CMT-U27.

### 2.13 *In vitro* T-cell mediated cytotoxicity and activation assay for anti-CD3-CMT-U27 cell

The target cells (anti-CD3-CMT-U27) were seeded in 96-well plates at a density of 1 × 10^4^ cells per well and allowed to adhere overnight. The following day, PBMCs were added as effector cells at various effector-to-target (E: T) ratios, corresponding to PBMC quantities of 1 × 10^5^, 5 × 10^4^, 2.5 × 10^4^, 1.25 × 10^4^, 0.625 × 10^4^, and 0.3125 × 10^4^ per well. Control wells included wild-type CMT-U27 cells co-cultured with PBMCs, target cells alone, and PBMCs alone. After 48-h co-culture, the culture supernatants were carefully collected and stored at −80 °C for subsequent canine IFN-*γ* analysis. The adherent cells were then gently washed twice with PBS to remove non-adherent cells and debris. To assess the remaining viable cells, they were fixed and stained with 0.5% crystal violet (Cat# C8470, Solarbio, China) in 25% methanol for 20 min. The plates were then rinsed thoroughly with tap water to remove excess dye and air-dried. The stained crystal violet was solubilized by adding a 3% glacial acetic acid (Cat#64-19-7, nj-reagent, China) to each well, and the absorbance was measured at 570 nm using a microplate reader.

### 2.14 Statistical analysis

Data are presented as mean ± standard deviation (SD). Statistical analyses were performed using GraphPad Prism 8 software (GraphPad Software, Boston, MA, USA). For comparisons between two groups, an unpaired, two-tailed Student’s *t*-test was used. Comparisons across three or more groups were conducted by one-way analysis of variance (ANOVA). A *p*-value of less than 0.05 was considered statistically significant.

## 3 Results

### 3.1 Generation of a rabbit anti-canine HAC19.1-specific monoclonal antibody

**Figure 1:**
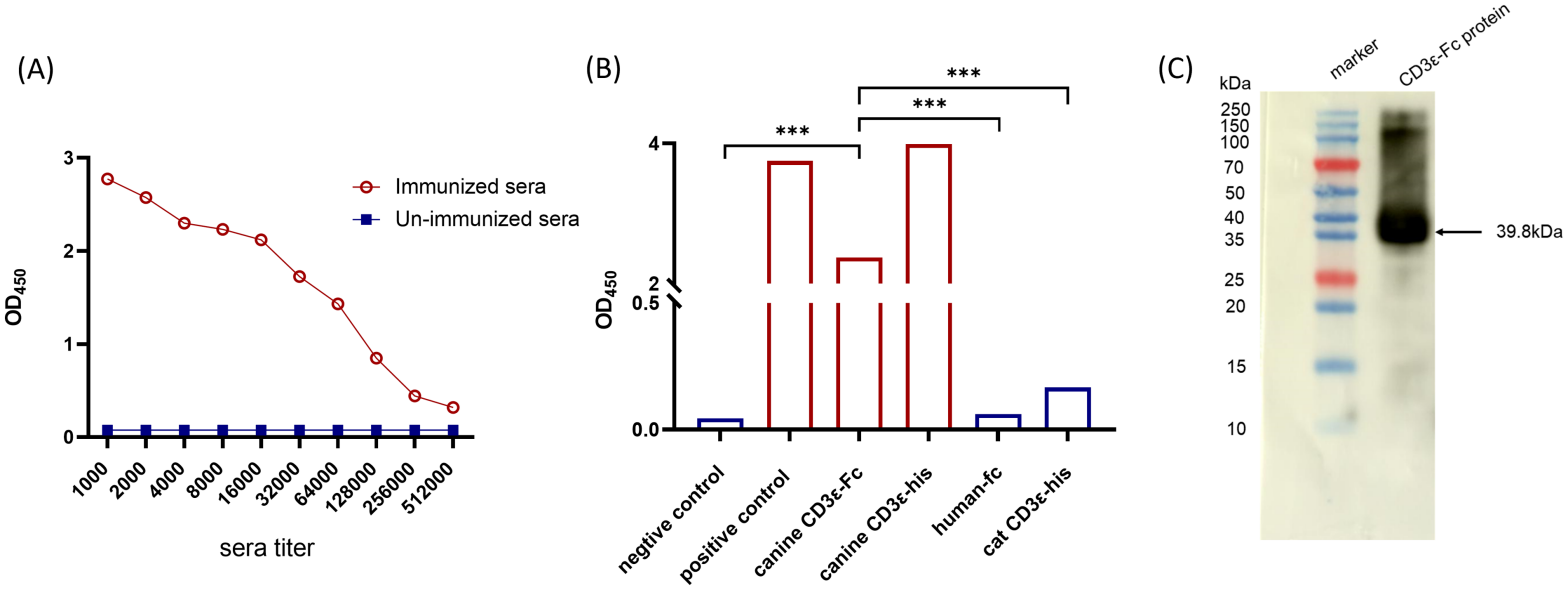
Generation of a rabbit anti-Canine CD3 Specific Monoclonal Antibody (HORCF-CD3.1). **(A)** Serum titer detection of canine CD3 antibody. All experiments were conducted in duplicate, and the results were reported as mean values. The immunized group (circles) showed a statistically significant difference (*p* < 0.01) compared to the non-immunized group (squares). **(B)** Validation of the binding between CD3ε mAb and CD3 protein by ELISA. All experiments were conducted in duplicate, and the results were reported as mean values. Compared to the negative control and human-Fc, a statistically significant difference (*p* < 0.001) was observed. **(C)** The CD3ε mAb specifically recognizes the CD3 protein in Western blot. The expected molecular weight of the CD3ε protein is approximately 39.8 kDa.

As shown in Figure 1A, serum analysis from immunized rabbits showed a high endpoint titer of canine CD3ε-specific antibodies, which was 1:512,000 by ELISA. This high titer of anti-canine CD3ε-specific antibodies (1,512,000) demonstrates strong immunogenicity of the canine CD3ε-Fc recombinant protein and confirms the efficacy of the immunization strategy. The ELISA results (Figure 1B) demonstrated that we successfully expressed of the HORCF-CD3.1 antibody, which could bind to both canine CD3ε-his and canine CD3ε-Fc proteins. The monoclonal antibody did not react with the human Fc tag or feline CD3ε-his protein, which is consistent with the expected outcome. This result proves that two critical properties. The mAb has high species specificity, The mAb selectively recognizes canine CD3ε epitopes without cross-reacting with feline homologs, suggesting minimal interference in multi-species experimental settings. The mAb has epitope localization, the ability to bind CD3ε regardless of the fusion tag (His or Fc) implies that the antibody targets the native conformational epitopes of canine CD3ε rather than tag-associated regions. This specificity is essential for applications requiring precise targeting of canine T cells. Western blot analysis further confirmed that the antibody exhibits strong immunogenicity and is capable of binding to the expressed CD3ε-Fc protein (Figure 1C). The robust expression in the chosen expression system (CHO cells) indicates optimized vector design, codon usage, and protein folding. The antibody’s ability to bind CD3ε-Fc demonstrates its recognition of conformational or linear epitopes, essential for downstream applications such as flow cytometry or therapeutic targeting. The absence of non-specific bands in the Western blot further supports the antibody’s high purity and specificity, minimizing off-target risks in functional assays.

### 3.2 HORCF-CD3.1 specifically binds to canine T cells

**Figure 2:**
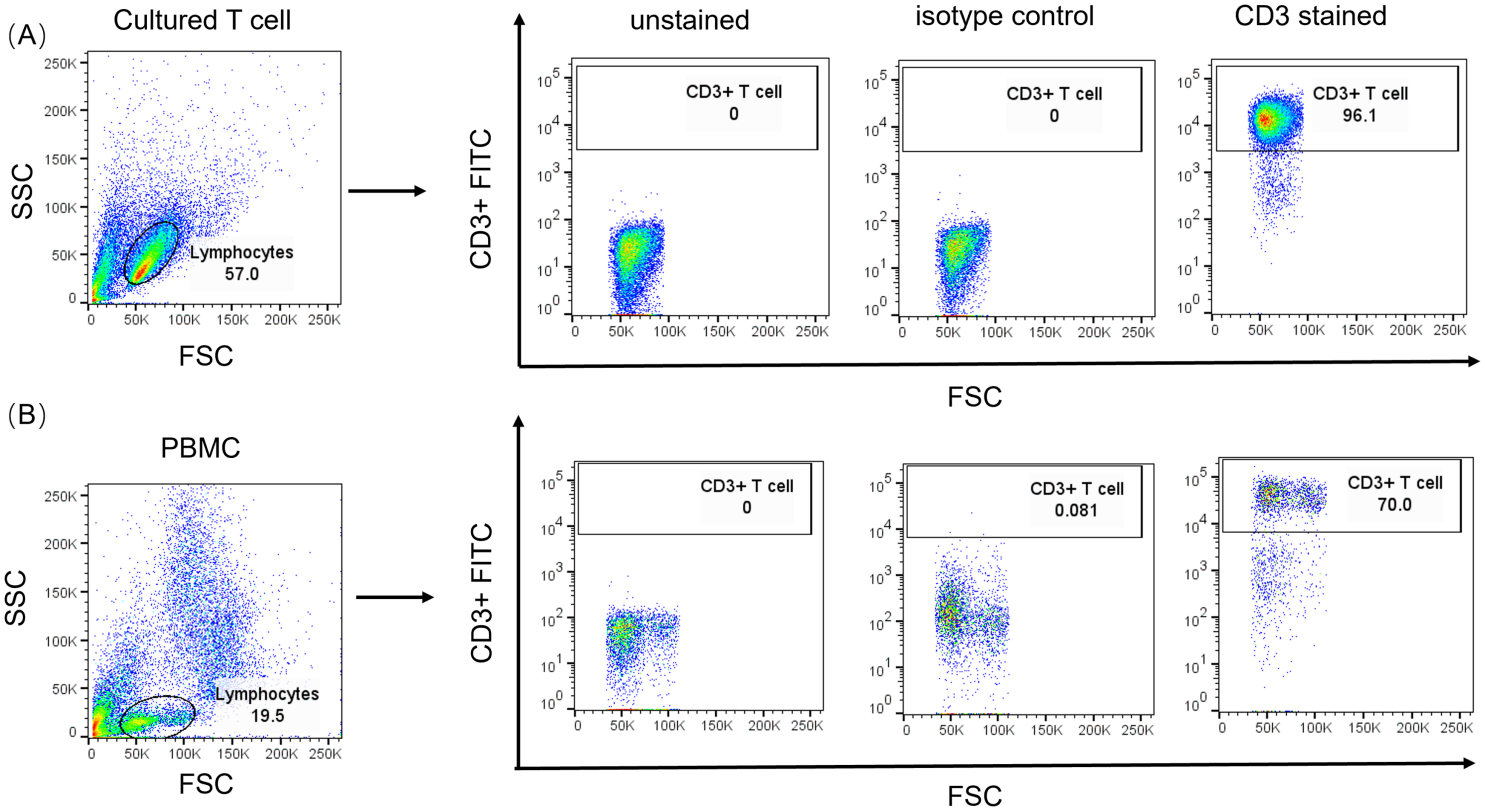
HORCF-CD3.1 demonstrates specific binding to the CD3 antigen present on the T cell surface. **(A)** Representative dot plots illustrating the forward scatter (FSC) versus side scatter (SSC) properties, with gating applied to the lymphocyte population, were shown. **(B)** The area within and above the rectangle represents positive results, while the area below the rectangle indicates negative results. **(A)** Represent the cultured T cells, while **(B)** denote the uncultured PBMCs.

Flow cytometric results demonstrated (Figure 2) that the monoclonal antibody demonstrated exclusive binding to T cell surface markers (CD3ε), with no observed cross-reactivity to B cells (CD19^+^) or monocytes (CD14^+^), indicative of its precise targeting capability. The distinct population segregation (e.g., clear differentiation between T cells and negative controls) confirmed the antibody’s applicability in flow cytometric analysis of complex biological samples, including whole blood and tumor-infiltrating lymphocytes, thereby significantly enhancing experimental reproducibility. The results indicate that the monoclonal antibody is suitable for flow cytometry applications and exhibits excellent specificity.

### 3.3 HORCF-CD3.1 could induce T cell activation

**Figure 3:**
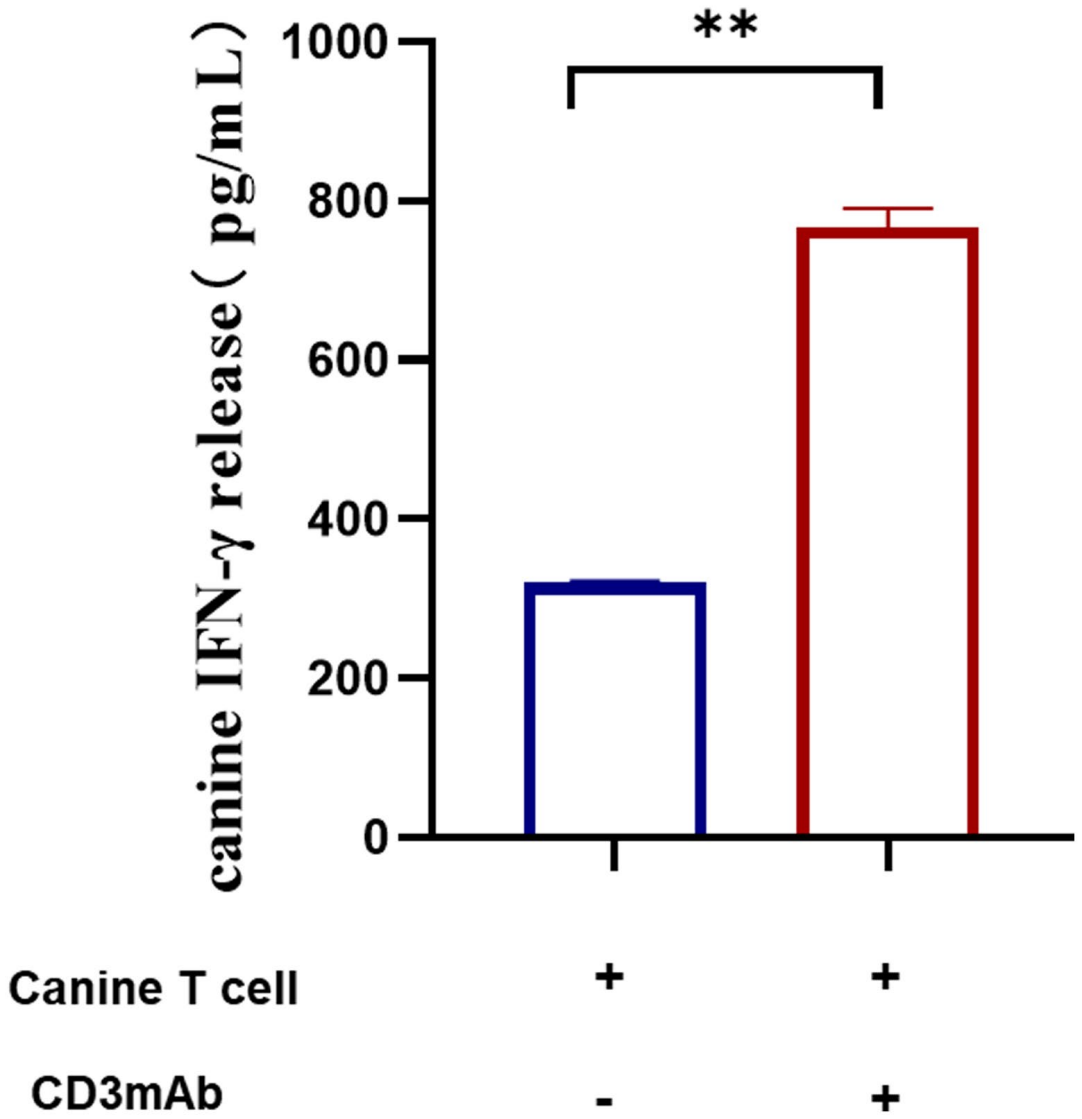
HORCF-CD3.1 could induce T cell activation. The evaluated data between the two groups showed statistically significant differences. An asterisk (*) indicates a significant (*p* < 0.05) difference between specific doses. Data were representative of three replicates experiments (mean ± SD of three technical replicates).

A significant increase in IFN-*γ* secretion was observed upon antibody stimulation, with the release level more than doubling compared to the unstimulated control (Figure 3). The increased secretion of IFN-*γ* enhances the body’s immune defense, particularly against viral infections and tumor growth. IFN-γ can promote Th1 cell differentiation and inhibit Th2 cell development, modulating the immune response. The phenomenon of IFN-γ secretion increase induced by antibody stimulation can be explored for its potential in disease treatment. In cancer immunotherapy, it is plausible that utilizing antibodies to enhance immune cell activity and IFN-γ production may lead to improved therapeutic efficacy.

### 3.4 Anti-CD3 conjugation on canine CMT-U27 cells induces T cell activation and cytotoxicity

**Figure 4:**
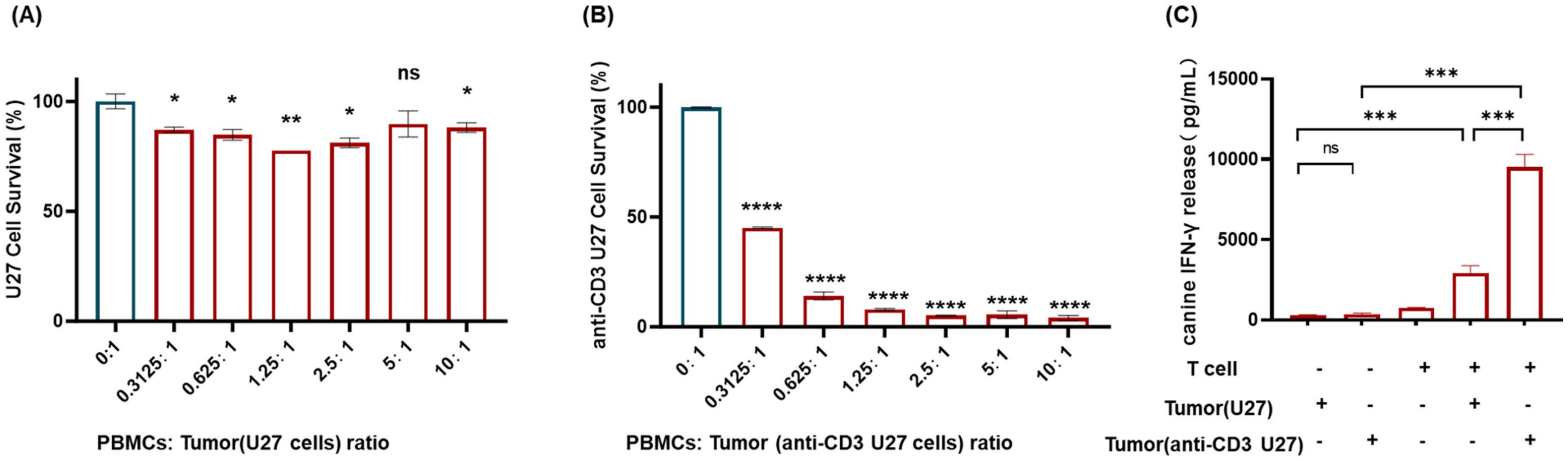
Anti-CD3 conjugation on canine CMT-U27 cells induces T cell activation and cytotoxicity. **(A)** Inhibition of proliferation of CMT-u27 cells following co-culture with increasing numbers of T cells, as determined by crystal violet assay. **(B)** Inhibition of proliferation of anti-CD3-conjugated CMT-U27 (anti-CD3-U27) cells following co-culture with increasing numbers of T cells, assessed by crystal violet assay. **(C)** IFN-*γ* secretion levels after co-culture of 100,000 T cells with either CMT-U27 or anti-CD3-U27 cells. The groups of CMT-U27 and anti-CD3-U27 cells alone (without T cells) were used as negative controls, respectively. Data are presented as mean ± SEM. Statistical significance was determined by Student’s *t*-test (*p* < 0.05 was considered significant).

The crystal violet assay was employed to assess the viability of anti-CD3-CMT-U27 canine tumor cells following co-culture with varying numbers of canine PBMCs. Results demonstrated a strong positive correlation between the number of PBMCs and the degree of anti-CD3-CMT-U27 cell death. The proliferation inhibition rate of anti-CD3-CMT-U27 cells increased proportionally with the increasing effector-to-target ratio. At a high E: T ratio (100,000 PBMCs), anti-CD3-CMT U27 cells were almost completely eradicated, showing near-total cell death compared to the control group (CMT-U27 co-cultured with PBMCs) (Figures 4A-B). To further evaluate T-cell activation within the PBMC population, the secretion of interferon-gamma (IFN-*γ*) was measured. Upon co-culture of 10,000 anti-CD3-CMT U27 cells with 100,000 PBMCs (E: T ratio of 10:1), a significant increase in IFN-γ concentration was detected compared to the control group (Figure 4C). The results indicate that ligation of the T-cell receptor by the anti-CD3 antibody potently activates T cells, leading to the direct lysis of target tumor cells.

## 4 Discussion

In this study, we employed prokaryotic and eukaryotic expression systems combined with single B-cell cloning technology. Using the eukaryotic-expressed CD3ε-Fc fusion protein as an immunogen, we successfully isolated antigen-specific memory B cells (MBCs) and generated the high-affinity rabbit-derived anti-canine CD3ε monoclonal antibody HORCF-CD3.1. The monoclonal antibody demonstrated exceptional functional properties. Western blot result confirmed that the HORCF-CD3.1 monoclonal antibody exhibited specific binding to eukaryotic-expressed canine CD3ε Fc protein. Flow cytometry revealed a remarkably high T-cell positivity rate of 98.1% in cultured T cells. Subsequent T-cell activation assays demonstrated that stimulation with the CD3 monoclonal antibody effectively induced robust T-cell activation and perfectly capable of killing tumor cells, accompanied by significant secretion of interferon-gamma (IFN-*γ*).

Therapeutic antibodies targeting CD3 hold dual potential in oncology and autoimmune diseases. For instance, drugs such as Teplizumab (anti-CD3 for type 1 diabetes) and Epcoritamab (CD3/CD20 bispecific antibody for lymphoma) have advanced in clinical application in humans (9, 10). Our study demonstrates that HORCF-CD3.1 is compatible with ELISA, Western blot, and flow cytometry, offering a standardized approach for monitoring T cell-related diseases in dogs. Furthermore, it can be utilized in the development of CD19-CD3 bispecific antibodies, enhancing the specificity of T cell-mediated tumor cell killing, in line with strategies used for human bispecific antibodies, while also demonstrating significant potential for precise detection and targeted therapy of T-cell lymphoma, as well as exhibiting promising applications in the treatment of autoimmune diseases (11–13). Compared to other models, the canine model serves as a vital tool in cancer research. On one hand, dogs spontaneously develop tumors and possess a fully intact immune system, which enables them to replicate key aspects of human cancer biology. On the other hand, companion dogs share a similar living environment with humans, and the etiology of tumors in dogs exhibits a higher degree of similarity to human tumors compared to other animals (14). Consequently, canine antibodies are not only applicable for the development of tumor models but also hold potential for the diagnosis and treatment of tumors. Furthermore, comparative studies across species could elucidate conserved versus divergent roles of CD3 signaling, potentially refining cross-species therapeutic strategies. However, a principal limitation of this study is the lack of *in vivo* data. The compelling *in vitro* evidence, while promising, necessitates validation in a living system to fully ascertain the antibody’s therapeutic potential. A paramount focus will be placed on evaluating critical efficacy endpoints, specifically the antibody’s ability to induce T cell expansion and its precise in vivo trafficking and persistence. Success in these areas will bridge our foundational findings to clinical relevance, thereby de-risking future translational development.

In conclusion, our study developed and validated a novel rabbit anti-canine CD3ε monoclonal antibody (HORCF-CD3.1) to provide a new tool for canine T cell-related research, which underscores the necessity of bridging species-specific research tools to accelerate both human and veterinary medical progress. These findings collectively underscore the therapeutic potential of this antibody in the development of novel immunotherapeutic strategies for precision cancer treatment. Future studies will therefore prioritize *in vivo* investigations in canine models.

## Data Availability

The original contributions presented in the study are included in the article/Supplementary material, further inquiries can be directed to the corresponding authors.

## Footnotes

^1^ https://www.uniprot.org/uniprotkb/P27597/entry

## References

[1] Notarangelo LD. Immunodeficiency and immune dysregulation associated with proximal defects of T cell receptor Signaling. Curr Opin Immunol. (2014) 31:97–101. doi: 10.1016/j.coi.2014.10.003, PMID: 25459000 PMC4254644

[2] Deng H Niu Z Zhang Z Zhang J Wang G Wang Y . Back on the scene: advances and challenges in Cd3-related drugs in tumor therapy. Drug Discov Today. (2022) 27:2199–208. doi: 10.1016/j.drudis.2022.04.019, PMID: 35489674

[3] Gupta S Rau RE Kairalla JA Rabin KR Wang C Angiolillo AL . Blinatumomab in standard-risk B-cell acute lymphoblastic Leukemia in children. N Engl J Med. (2025) 392:875–91. doi: 10.1056/NEJMoa2411680, PMID: 39651791 PMC11864901

[4] London CA Gardner H Zhao S Knapp DW Utturkar SM Duval DL . Leading the pack: best practices in comparative canine cancer genomics to inform human oncology. Vet Comp Oncol. (2023) 21:565–77. doi: 10.1111/vco.12935, PMID: 37778398 PMC12065084

[5] Sakthikumar S Warrier M Whitley D Facista S Adkins J Aman S . Genomic analysis across 53 canine cancer types reveals novel mutations and high clinical actionability potential. Vet Comp Oncol. (2024) 22:30–41. doi: 10.1111/vco.12944, PMID: 38053317

[6] Sorenmo KU Harwood LP King LG Drobatz KJ. Case-control study to evaluate risk factors for the development of sepsis (neutropenia and fever) in dogs receiving chemotherapy. J Am Vet Med Assoc. (2010) 236:650–6. doi: 10.2460/javma.236.6.650, PMID: 20225976

[7] Simon D Schoenrock D Baumgärtner W Nolte I. Postoperative adjuvant treatment of invasive malignant mammary gland tumors in dogs with doxorubicin and docetaxel. J Vet Intern Med. (2006) 20:1184–90. doi: 10.1892/0891-6640(2006)20[1184,patimm]2.0.co;2 17063714

[8] Dezfulian MH Kula T Pranzatelli T Kamitaki N Meng Q Khatri B . Tscan-ii: a genome-scale platform for the De novo identification of Cd4(+) T cell epitopes. Cell. (2023) 186:5569–5586.e21. doi: 10.1016/j.cell.2023.10.024, PMID: 38016469 PMC10841602

[9] Herold KC Bundy BN Long SA Bluestone JA DiMeglio LA Dufort MJ . An anti-Cd3 antibody, teplizumab, in relatives at risk for type 1 diabetes. N Engl J Med. (2019) 381:603–13. doi: 10.1056/NEJMoa1902226, PMID: 31180194 PMC6776880

[10] Li T Gibiansky L Parikh A van der Linden M Sanghavi K Putnins M . Population pharmacokinetics of epcoritamab following subcutaneous administration in relapsed or refractory B cell non-Hodgkin lymphoma. Clin Pharmacokinet. (2025) 64:127–41. doi: 10.1007/s40262-024-01464-2, PMID: 39708278

[11] Bekyarova AI Kobakova I Spasova S. A case of abscessing ileocecal monomorphic epitheliotropic intestinal T-cell lymphoma. Cureus. (2024) 16:e72169. doi: 10.7759/cureus.72169, PMID: 39583401 PMC11584210

[12] Briones AC Megino RF Marin AV Chacón-Arguedas D García-Martinez E Balastegui-Martín H . Nonsense Cd247 mutations show dominant-negative features in T-cell receptor expression and function. J Allergy Clin Immunol. (2024) 154:1022–32. doi: 10.1016/j.jaci.2024.06.019, PMID: 38992472

[13] Shouse G. Bispecific antibodies for the treatment of hematologic malignancies: the magic is T-cell redirection. Blood Rev. (2025) 69:101251. doi: 10.1016/S0021-9258 39617677

[14] Overgaard NH Fan TM Schachtschneider KM Principe DR Schook LB Jungersen G. Of mice, dogs, pigs, and men: choosing the appropriate model for Immuno-oncology research. ILAR J. (2018) 59:247–62. doi: 10.1093/ilar/ily014, PMID: 30476148

