# Supplementary material for: Preparation and functional validation of rabbit anti-canine CD3ε monoclonal antibody
Source: Front Vet Sci. 2025 Dec 4;12:1612069. doi: 10.3389/fvets.2025.1612069 (PMC12711479; doi:10.3389/fvets.2025.1612069)
Supplement: Supplementary file 1 [file Data_Sheet_1.pdf]

Supplementary Data 1

Canine CD3ε protein sequences

CD3 original protein sequence（Uniprot/P27597）

MQSRNLWRILGLCLLSVGAWG(signal peptide)QDEDFKASDDLTSISPEKRFKVSISGTEVV
VTCPDVFGYDNIKWEKNDNLVEGASNRELSQKEFSEVDDSGYYACYADSIKEKSYLYLRA
RVCANCIEVNLMAVVTIIVADICLTLGLLLMVYYWSKTRKANAKPVMRGTGAGSRPRGQ
NKEKPPPVPNPDYEPIRKGQQDLYSGLNQRGI

CD3-Fc eukaryotic expression protein sequence

MQSRNLWRILGLCLLSVGAWG(signal peptide)QDEDFKASDDLTSISPEKRFKVSISGTEVV
VTCPDVFGYDNIKWEKNDNLVEGASNRELSQKEFSEVDDSGYYACYADSIKEKSYLYLRA
RVCANCIEVHTCPPCPAPELLGGPSVFLFPPKPKDTLMINLEPKSCDKTSRTPEVTCVVVDV
SHEDPEVKFNWYVDGVEVHNAKTKPREEQYNSTYRVVSVLTVLHQDWLNGKEYKCKVS
NKALPAPIEKTISKAKGQPREPQVYTLPPSRDELTKNQVSLTCLVKGFYPSDIAVEWESNGQ
PENNYKTTPPVLDSDGSFFLYSKLTVDKSRWQQGNVFSCSVMHEALHNHYTQKSLSLSPG
K

CD3-his prokaryotic expression protein sequence

QDEDFKASDDLTSISPEKRFKVSISGTEVVVTCPDVFGYDNIKWEKNDNLVEGASNRELSQ
KEFSEVDDSGYYACYADSIKEKSYLYLRARVCANCIEVNLMAVVTIIVADICLTLGLLLMV
YYWSKTRKANAKPVMRGTGAGSRPRGQNKEKPPPVPNPDYEPIRKGQQDLYSGLNQRGI
HHHHHH
