# Supplementary material for: Preparation and functional validation of rabbit anti-canine CD3ε monoclonal antibody
Source: Front Vet Sci. 2025 Dec 4;12:1612069. doi: 10.3389/fvets.2025.1612069 (PMC12711479; doi:10.3389/fvets.2025.1612069)
Supplement: Supplementary file 2 [file Supplementary_file_1.zip › Source Date/Figure 1/figure 1B/Instructions.docx]

Through single B cell sorting technology, we isolated individual B cells and subsequently identified 42 antibody clones via comprehensive screening. These antibodies were systematically evaluated for their binding capacity to both the Fc fragment and the canine CD3E-Fc recombinant protein, resulting in the identification of 25 functional antibodies that met predefined selection criteria. Subsequent validation experiments demonstrated that all 25 selected antibodies exhibited specific binding to canine CD3E-his protein, while showing no detectable interaction with feline CD3E-his protein under identical experimental conditions.
