# Supplementary material for: Preparation and functional validation of rabbit anti-canine CD3ε monoclonal antibody
Source: Front Vet Sci. 2025 Dec 4;12:1612069. doi: 10.3389/fvets.2025.1612069 (PMC12711479; doi:10.3389/fvets.2025.1612069)
Supplement: Supplementary file 2 [file Supplementary_file_1.zip › Source Date/Figure 2/Instruction.docx]

The flow cytometry data analysis was performed using a hierarchical gating approach to ensure population specificity:

1. PBMC-derived T cell identification: Primary lymphocytes within PBMCs were initially defined by gating on forward scatter area (FSC-A) versus side scatter area (SSC-A) to exclude debris and granulocytes (threshold: FSC-A:320, SSC-A :280)

T lymphocyte populations were subsequently resolved through CD3 selection using CD3 monoclonal and fluorescence-conjugated goat anti-rabbit 488 antibodies, and compensation matrices validated through un-stained and isotype control.

1. Cultured T cell discrimination: Cultured T cell viability was assessed through FSC/SSC profiling, all gating thresholds were established based on unstained control and isotype control cohorts and maintained consistently across experimental replicates.
