# Supplementary material for: Preparation and functional validation of rabbit anti-canine CD3ε monoclonal antibody
Source: Front Vet Sci. 2025 Dec 4;12:1612069. doi: 10.3389/fvets.2025.1612069 (PMC12711479; doi:10.3389/fvets.2025.1612069)
Supplement: Supplementary file 2 [file Supplementary_file_1.zip › Source Date/Figure 4/Analysis.pptx]

## Slide 1
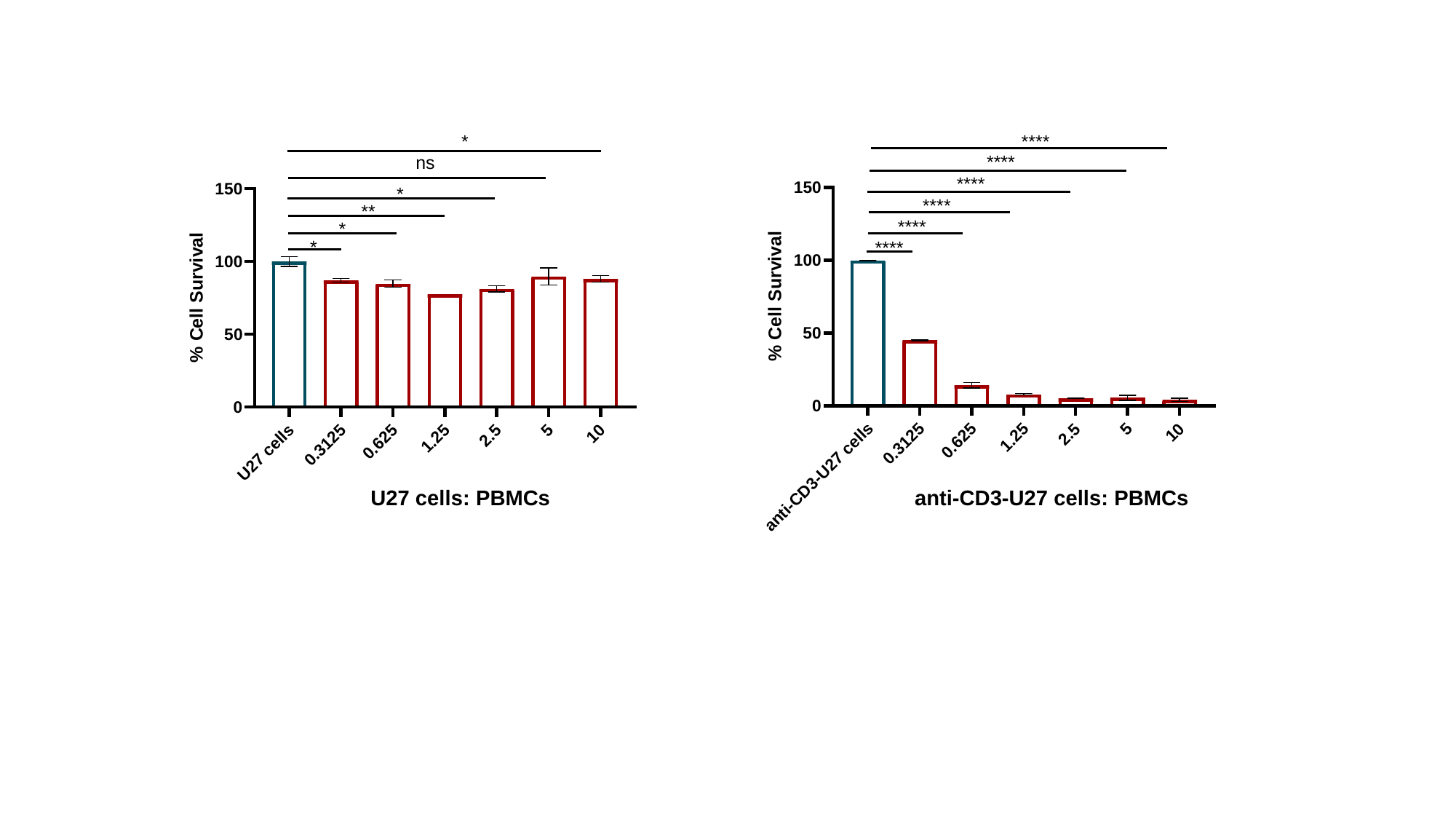

U27 cells: PBMCs
anti-CD3-U27 cells: PBMCs

## Slide 2
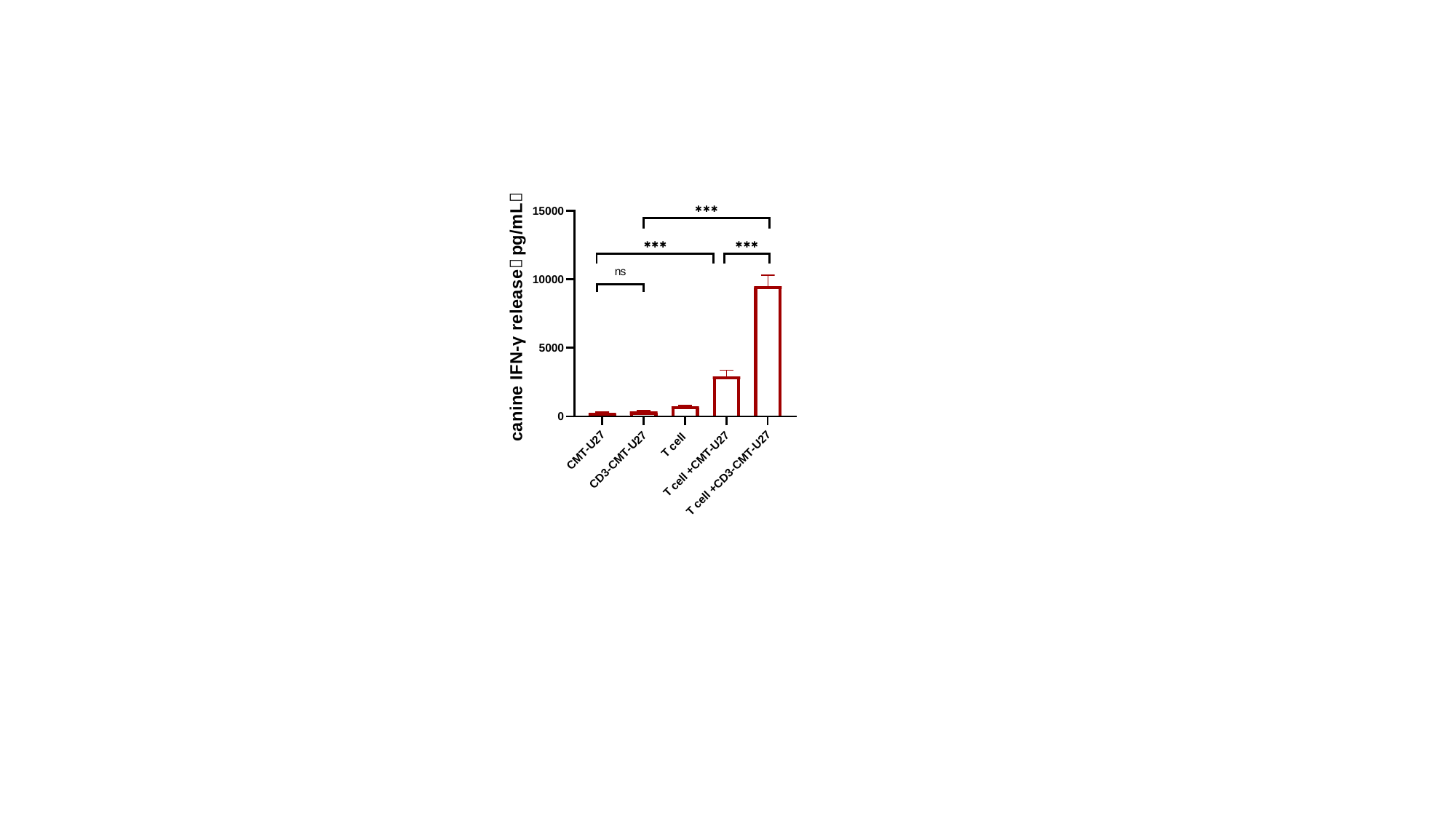

## Slide 3
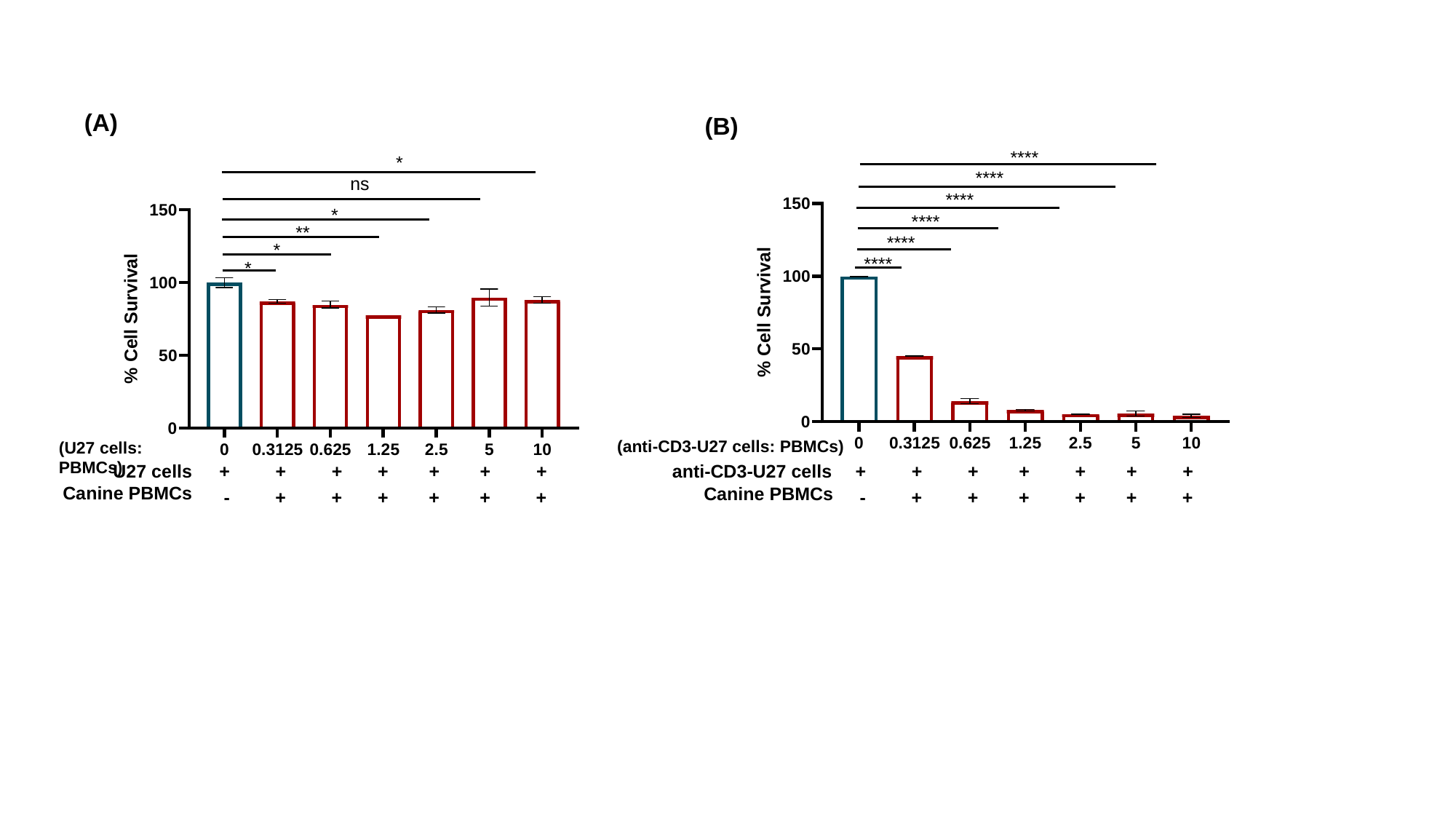

(A)
(U27 cells: PBMCs)
U27 cells
 + + + + + + +
Canine PBMCs
 - + + + + + +
(B)
(anti-CD3-U27 cells: PBMCs)
anti-CD3-U27 cells
 + + + + + + +
Canine PBMCs
 - + + + + + +

## Slide 4
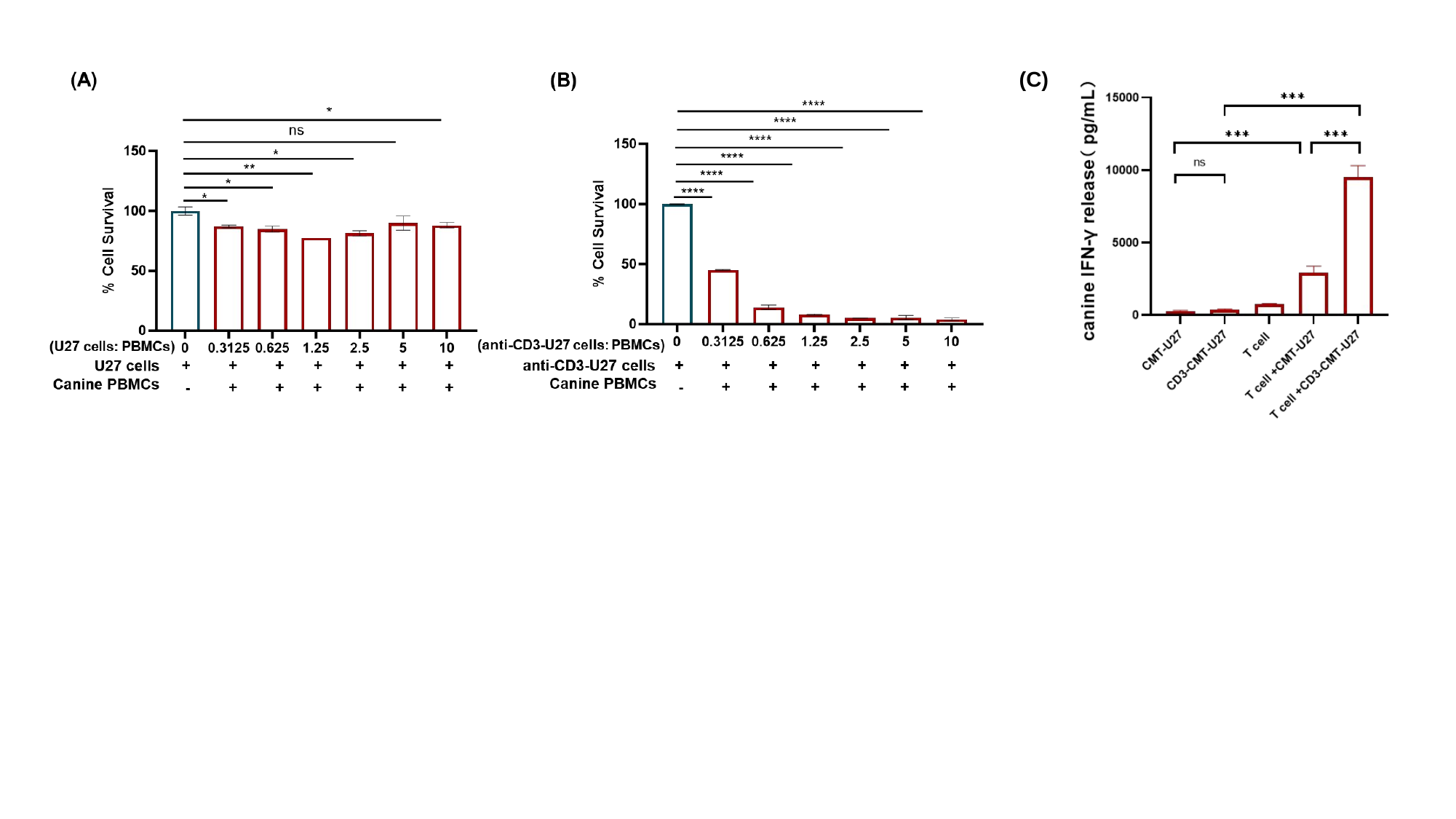

(C)
